# Crystal structure of hyperthermophilic esterase EstE1 and the relationship between its dimerization and thermostability properties

**DOI:** 10.1186/1472-6807-7-47

**Published:** 2007-07-12

**Authors:** Jung-Sue Byun, Jin-Kyu Rhee, Nam Doo Kim, JeongHyeok Yoon, Dong-Uk Kim, Eunhee Koh, Jong-Won Oh, Hyun-Soo Cho

**Affiliations:** 1 Department of Biology, Yonsei University, 134 Shinchon-dong, Seodaemun-gu, Seoul 120-749, Korea; 2 Department of Biotechnology, Yonsei University, 134 Shinchon-dong, Seodaemun-gu, Seoul 120-749, Korea; 3 Drug Discovery R&D Center, Equispharm Co., Ltd., Sungnam 463-020, Korea

## Abstract

**Background:**

EstE1 is a hyperthermophilic esterase belonging to the hormone-sensitive lipase family and was originally isolated by functional screening of a metagenomic library constructed from a thermal environmental sample. Dimers and oligomers may have been evolutionally selected in thermophiles because intersubunit interactions can confer thermostability on the proteins. The molecular mechanisms of thermostabilization of this extremely thermostable esterase are not well understood due to the lack of structural information.

**Results:**

Here we report for the first time the 2.1-Å resolution crystal structure of EstE1. The three-dimensional structure of EstE1 exhibits a classic α/β hydrolase fold with a central parallel-stranded beta sheet surrounded by alpha helices on both sides. The residues Ser154, Asp251, and His281 form the catalytic triad motif commonly found in other α/β hydrolases. EstE1 exists as a dimer that is formed by hydrophobic interactions and salt bridges. Circular dichroism spectroscopy and heat inactivation kinetic analysis of EstE1 mutants, which were generated by structure-based site-directed mutagenesis of amino acid residues participating in EstE1 dimerization, revealed that hydrophobic interactions through Val274 and Phe276 on the β8 strand of each monomer play a major role in the dimerization of EstE1. In contrast, the intermolecular salt bridges contribute less significantly to the dimerization and thermostability of EstE1.

**Conclusion:**

Our results suggest that intermolecular hydrophobic interactions are essential for the hyperthermostability of EstE1. The molecular mechanism that allows EstE1 to endure high temperature will provide guideline for rational design of a thermostable esterase/lipase using the lipolytic enzymes showing structural similarity to EstE1.

## Background

Lipolytic enzymes, including esterases or carboxylesterases (EC 3.1.1.1) and lipases (EC 3.1.1.3), catalyze the stereospecific hydrolysis, transesterification, and conversion of a variety of amines and primary and secondary alcohols [1-3]. Esterases and carboxylesterases hydrolyze partly-soluble fatty acid esters with acyl chain lengths of less than 10 carbon atoms [4], whereas lipases act on water-insoluble long-chain triglycerides. Many lipases and esterases have been isolated from a variety of microorganisms, animals, plants, and metagenomes [4,5].

Thermostable esterases/lipases originate from thermophiles. Their extraordinary thermodynamic stability allows these enzymes to function in organic solvents, and at elevated temperatures that approach or exceed 100°C [5,6]. Despite the many biotechnological and industrial applications of thermostable lipolytic enzymes, only less than one dozen of them have been isolated from thermophiles, hyperthermophiles, and metagenomes from thermal environments [4-6]. We recently reported a new hormone-sensitive esterase/lipase (HSL) family [7] hyperthermostable esterase, EstE1, which was isolated by functional screening of a metagenomic DNA librariy constructed from a thermal environment sample [5]. EstE1 is composed of 311 amino acid residues with a calculated molecular weight of approximately 34 kDa. It exhibits an esterase activity on short chain acyl derivatives of length C4–C6 at temperatures of 30 to 90°C, and is stable at temperatures exceeding 90°C [5,8] The amino acid sequence of EstE1 is significantly similar to other thermostable HSL-family esterases, including an esterase from hyperthermophilic archaeon *Pyrobaculum calidifontis *(64%) [9], the esterase AFEST from hyperthermophilic archaeon *Archeoglobus fulgidus *(57%) [10], and the esterase EST2 from thermophilic bacterium *Alicyclobacillus acidocaldarius *(51%) [8] Recently, the three-dimensional (3D) structure of EstE1 was predicted by homology modeling using the AFEST esterase as a reference [8]. Structure and sequence-based analyses for the thermostability determinants of EstE1 identified multiple intramolecular ion-pair networks and hydrophobic interactions critical for the thermostability of EstE1 [8].

Dimers and oligomers are often the functional form of proteins and may have been evolutionarily selected to confer thermostability on the proteins in thermopiles because subunit associations can result in extra stabilization of the proteins. Indeed, many enzymes from thermophilic organisms are capable of forming higher order oligomers [11-15], but only a few have been studied in detail to determine what interfacial interactions are responsible for their thermostability. Here we report for the first time the crystal structure of EstE1 at 2.1-Å resolution. By structural and functional analysis of the thermostability determinants of EstE1, we found that EstE1 dimerization through hydrophobic interactions is the major contributor for the hyperthermostability of EstE1.

## Results

### Structure of the EstE1 monomer

The 2.1-Å resolution crystal structure of the selenomethionine derivative of EstE1 was determined in space group P4_1_2_1_2 (unit cell dimensions of a = b = 73.71 Å, c = 234.23 Å, and α = β = γ = 90°) with two independent molecules per asymmetric unit, by single wavelength anomalous dispersion (SAD) method. Several cycles of simulated annealing, minimization, and *B *group refinement using the program CNS [16], followed by manual model rebuilding, reduced the R values for all the data in the resolution range of 50.0–2.1 Å. The R_factor _of the present model is 22.3% with an R_free _value of 26.4%. The crystallographic statistics for data collection and refinement are summarized in Table 1.

**Table 1:** Data collection and refinement statistics

	**SAD**	**Native**
**Data collection**		
Resolution (Å)	30–2.3	50–2.1
Wavelength (Å)	0.9792	0.9795
Total reflections	1,996,840	1,197,490
Unique reflections	29,848	38,527
Completeness (%)	99.8 (99.8)^*a*^	99.9 (100)^*a*^
R_sym _(%)^*b*^	11.9 (32.2)	13.7 (64.3)
Average *I*/σ(*I*)	30.4 (6.7)	17.0 (3.1)
**Refinement **		
Resolution (Å)	50–2.1	
R_cryst _(%)^*c*^	22.3	
R_free _(%)^*d*^	26.4	
Protein atoms	4,469	
Water molecules	138	
RMS deviations		
Bond lengths (Å)	0.0095	
Bond angles (°)	1.50	
Ramachandran plot (%)^*e*^		
Most favored	92.0	
Additionally allowed	7.2	
Generally allowed	0.8	
Disallowed	0	

^*a*^Values in parentheses are for the highest-resolution shell (2.38–2.30 Å for SAD and 2.18–2.10 Å for native).

^*b*^R_sym _= Σ|(*I*_*hkl*_) - <*I*>|/Σ(*I*_*hkl*_), where *I*_*hkl *_is the integrated intensity of a given reflection and <*I*> is the mean intensity of symmetry equivalents.

^*c*^R_cryst _= Σ||*F*_obs_| - |*F*_*calc*_||/Σ|*F*_*obs*_|, where *F*_obs _and *F*_*calc *_are the observed and calculated structure factors, respectively.

^*d*^R_free _is the R-factor calculated using a randomly selected 5% of reflection data withheld from the refinement.

^*e *^Calculated with the program PROCHECK [36].

**Figure 1:**
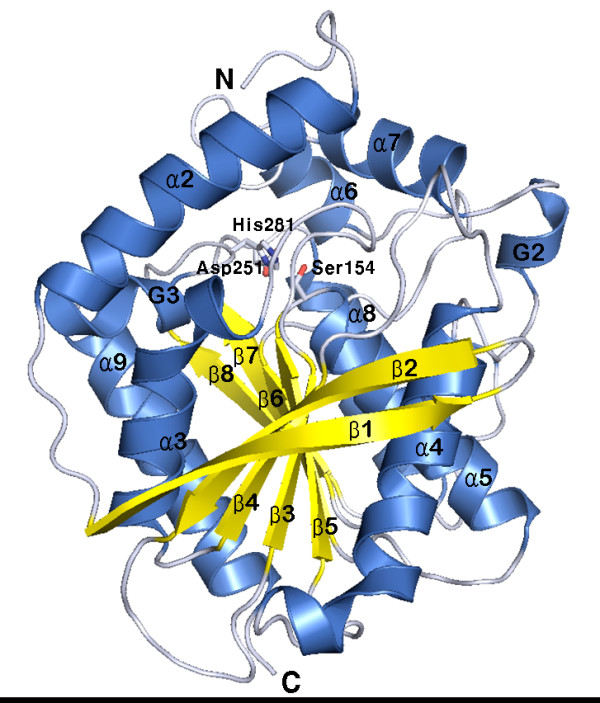
**Three-dimensional structure of EstE1**. A ribbon diagram of EstE1 shows the eight α-helices and eight β-strands that form a classical α/β hydrolase fold [17]. The α-helical segments and β-strands are shown in blue and yellow, respectively. G2 and G3 represent 3_10_-helices. Helix α1 is not shown because of its disordered electron map. The catalytic triad containing residues Ser154, Asp251, and His281, are shown in stick representation. N and C denote the N and C termini, respectively.

The final structure of EstE1 displayed a nearly ellipsoidal shape with approximate dimensions of 46 Å × 47 Å × 57 Å (Figure 1). It has the classical features of an α/β hydrolase fold [17] consisting of a central eight-stranded mixed β-sheet surrounded by the five helices α3 (Asp93-Ser103), α4 (Thr122-Leu141), α5 (Ala155-Ser170), α8 (Leu253-Ser267), and α9 (Asp291-Leu308). The electron density of the N-terminal 16 residues, which is expected to include the α1 (Lys8-Arg13) helix, was disordered. Strands β1 and β3 – β8 are parallel but the β2 strand is antiparallel to the others. The β1 and β8 strands are rotated approximately 90° because β2–β7 sheets are in the counter-clockwise direction starting from the position of the β1 strand and extending to that of the β8 strand. These β strands form an α/β fold core domain, which is the canonical architecture of an α/β hydrolase [17]. EstE1 also contains a similar cap domain present in other members of the HSL family [18-20]. The cap domain consists of two separated helical regions (containing residues 1–41 and 193–218) and locates to the upper region of the central β-sheet. The helices α1 (not shown in Figure 1; residues 9–14), α2 (residues 23–41), α6 (residues 193–200), α7 (residues 208–218), two 3_10_-helices (G2 and G3), and several random coils between these helices constitute the cap domain.

### Active site architecture: catalytic triad and substrate-binding pocket

The activity of α/β hydrolases primarily depends on a catalytic triad formed by Ser, His, and Asp/Glu residues [17,21,22]. The active site of EstE1 also resides in a canonical position (Figure 1) inferred from known structures of α/β hydrolases [17]. The key nucleophile Ser154, which is found in a highly conserved Gly-X-Ser-X-Gly pentapeptide (X denotes any amino acid) [10,20], locates to the apex of the nucleophilic elbow. The nucleophile backbone φ and ψ angles for Ser154 lie in an unfavorable region of the Ramanchandran plot (φ = 59° and ψ = -121°) because of the sharpness of the turn connecting the β5 strand and α5 helix. A hydrogen bond (3.1 Å) between the OG atom of Ser154 and NE2 atom of His281 stabilizes the conformation of the nucleophile Ser154. The proton carrier residues His281 and Asp251 that form the charge-relay network locate to the carboxyl edge of β-strands 7 and 8, respectively. The side-chains of His281 and Asp251 are stabilized by a network of hydrogen bonds formed by the ND1 atom of His281 and the OD2 atom of Asp251 (2.7 Å), the NE2 atom of His281 and the OG atom of Ser154 (3.1 Å), the OD1 atom of Asp251 and the NH atom of Leu253 (2.8 Å), and the O atom of Asp251 and the N atom of Arg254 (3.0 Å).

The substrate-binding pocket of EstE1 extends approximately 16 Å from the protein surface to the catalytic Ser154 residue. The deep hydrophobic cleft is funnel-shaped and its entrance is surrounded by three α-helices (α2, α6, and α7), the 3_10_-helix G3 (Phe283–Phe289), and the loop regions (Ile14-Ser22 and Ala201-Pro207). The sequence motif His-Gly-Gly-Gly (residues 80–83), which is conserved in the HSL family [20], is upstream of the active site. It is involved in hydrogen bonding for stabilization of the oxyanion hole formed by Gly82, Gly83, and Ala155. Residue Gly81 is fixed by hydrogen bonds between its amino group and the OD1 atom of Asp153 (3.1 Å), and between the carbonyl group of Gly81 and the ND1 atom of His30 (2.6 Å).

### Hydrophobic interactions essential for the dimerization of EstE1

**Figure 2:**
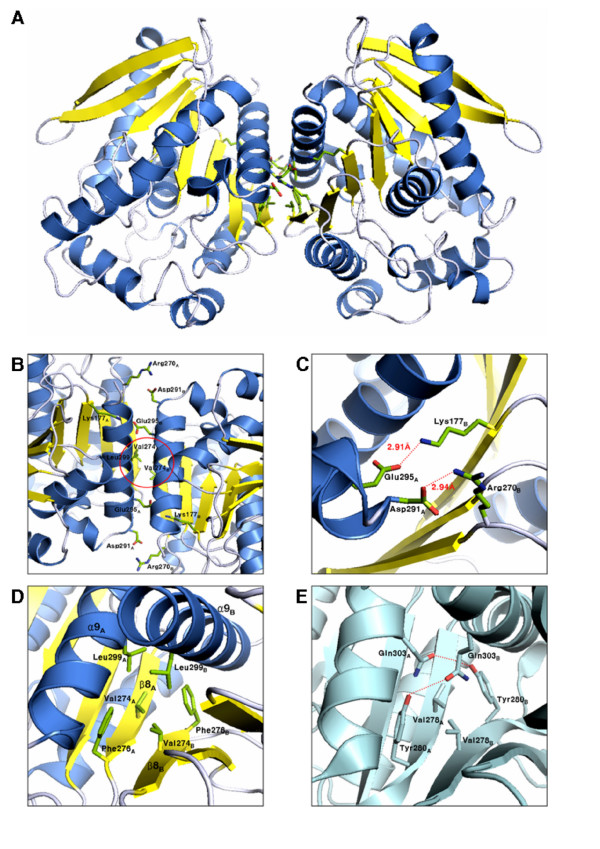
**Structure of the EstE1 dimer**. (A) The EstE1 dimer viewed along the crystallographic two-fold axis. Residues involved in hydrophobic interactions are shown in stick representation. Val274, Phe276, and Leu299 are involved in the hydrophobic interactions. Arg270-Asp291 and Lys177-Glu295 form salt bridges. (B) The centrosymmetric conformation of the interface between the two monomers, consisting of centric hydrophobic interactions (red circle) and salt bridges. (C) A detailed view of the salt bridges that support the dimeric conformation of EstE1. The side chain of Arg270_B _on the loop between the α8 helix and the β8 strand forms a salt bridge with Asp291_A _on the α9 helix. An additional salt bridge is formed between the side chains of Lys177_B _on the β6 strand and Glu295_A _on the α9 helix. (D) A detailed view of the hydrophobic interaction interface observed in the EstE1 dimer. The hydrophobic core residues (Leu299 on the α9 helix, and Phe276 and Val274 on the β8 strand) are indicated. (E) A detailed view of the interface observed in a current AFEST dimer model [19]. Dimeric interactions of AFEST are supported by hydrogen bonds between Tyr280 and Gln303, and by a weak hydrophobic interaction through Val278.

EstE1 exists as a dimer in solution. An EstE1 dimer has 2-fold crystallographic symmetry and displays an extensive dimeric interface spanning over 128 Å^2 ^(Figure 2A). Assuming two molecules comprise an asymmetric unit, the Matthews coefficient *V*_M _was calculated to be 2.2 Å^3^/Da, which corresponds to a solvent content of 44.1%. EstE1 dimerization is primarily mediated by hydrophobic interactions in the middle of the interface and is assisted by salt bridges at both ends of the interface (Figure 2B). The dimeric interface is characterized by the formation of β-sheet between the two monomers by an antiparallel association between two C-terminal β8 strands. We identified two salt bridges, Lys177 (on the β6 strand)-Glu295 (on the α9 helix) and Arg270 (on the loop between α8 helix and β8 strand)-Asp291 (on the α9 helix) (Figure 2C). These interactions exhibit centrosymmetric configuration toward the center of the interface. We also identified hydrophobic interactions between Val274 (on the β8 strand), Phe276 (on the β8 strand), and Leu299 (on the α9 helix) (Figure 2D).

**Figure 3:**
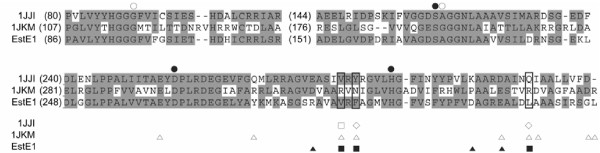
**Amino acid sequence alignment of EstE1 and its homologous HSL-family hyperthermophilic and mesophilic esterases**. Amino acid sequence of EstE1 was aligned with those of the hyperthermophilic carboxyesterase AFEST (Protein Data Bank [PDB] code 1JJI) from archaeon *Archaeoglobus fulgidus *and the mesophilic brefeldin A esterase (BFAE) from *Bacillus subtilis *(PDB code 1JKM). The regions encompassing EstE1 dimerization motifs and the sequence blocks showing the amino acids involved in the formations of the catalytic triad and oxyanion hole are presented. Identical and similar residues have a grey background. Symbols: ●, amino acids forming a catalytic triad; ○, amino acids involved in oxynion hole formation; □ and ▯, amino acid residues involved in hydrophobic and ionic interactions at 1JJI dimeric interface, respectively; ▯, amino acid residues involved in ionic interactions at 1JKM dimeric interface; ■ and ▲ amino acid residues involved in hydrophobic and ionic interactions at EstE1 dimeric interface, respectively. Amino acid sequence alignment was performed as described previously [5].

**Figure 4:**
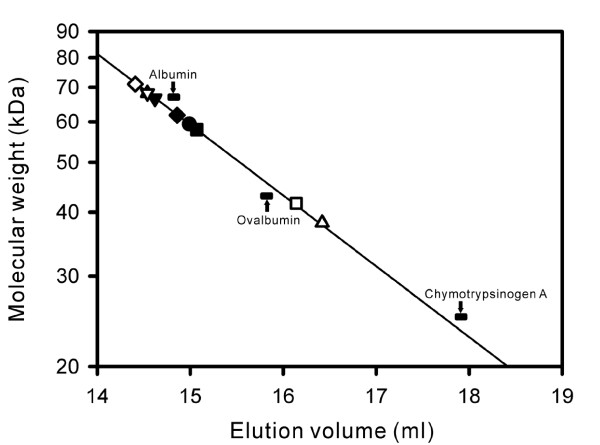
**Analysis of wild-type and mutant EstE1 by gel filtration chromatography**. Purified EstE1 proteins were loaded onto a Superdex 200 column and eluted as described in Methods. Estimated molecular weights of wild-type EstE1 (●), EstE1_F276A _(■), EstE1_F276E _(□), EstE1_V274A _(▲), EstE1_V274A/F276A _(▯), EstE1_L299D _(▼), EstE1_R270A _(▯), EstE1_E295A _(▯), and EstE1_R270A/E295A _(▯) are presented. Molecular mass standards (albumin, 67 kDa; ovalbumin, 43 kDa; and chymotrypsinogen A, 25 kDa) were subjected to the same process, and their migration is indicated.

The residues responsible for dimerization of EstE1 subunits differ from those of AFEST. A previous study showed that intermolecular contacts present in AFEST consisted of hydrogen bonds and salt bridges [19]. Amino acid sequence alignment revealed that Val274, Phe276, and Leu299 of EstE1 correspond to Val278, Tyr280, and Gln303 of AFEST, respectively (Figure 3), but these residues in AFEST did not participate in similar hydrophobic interactions as observed in EstE1. Instead, hydrogen bonds between Tyr280_A_-Gln303_B _and Tyr280_B_-Gln303_A_, and weak hydrophobic interactions between Val278_A _and Val278_B_, appear to contribute to the intermolecular interactions observed in AFEST (Figure 2E). To verify the roles of the hydrophobic interactions and salt bridges identified in EstE1 dimer interface, eight EstE1 mutant proteins containing single or multiple amino acid substitutions were created. Five EstE1 mutants had disruptions in hydrophobic interactions. Val274 was replaced with Ala to generate EstE1_V274A_. Phe276 was replaced with Ala and Glu to generate EstE1_F276A _and EstE1_F276E_, respectively. Leu299 was changed to Asp in EstE1_L299D_. Both Phe276 and Val274 were changed to Ala in EstE1_V274A/F276A_. In addition, three EstE1 mutants containing changes in the residues involved in the salt bridges were generated. Arg270 and Glu295 residues were replaced with Ala to generate EstE1_R270A _and EstE1_E295A_, respectively. Both of these amino acids were changed to Ala to generate EstE1_R270A/E295A_. All of these mutants were expressed in *E. coli *and purified to near-homogeneity from cell extracts that were not heat-treated, using Ni-affinity chromatography as described previously [8]. Analysis of the molecular weight of the purified mutant proteins by gel filtration chromatography (GFC) revealed that EstE1_F276E _and EstE1_V274A/F276A _converted to a monomer of approximately 42 and 38 kDa, respectively, while wild-type EstE1 and other mutants were the size of a dimer with a molecular weight of ~60 kDa (Figure 4). EstE1_F276A _remained as a dimer whereas a single mutation of Phe to Glu in EstE1_F276E _completely abolished dimerization. This is likely due to the fact that Ala has a similar hydrophobicity scale as that of Phe such that EstE1 molecules maintained their hydrophobic interactions, while change of the Phe to hydrophilic residue Glu did not permit the formation of hydrophobic interactions with Val274 in another molecule of EstE1. This result underscores the critical role of hydrophobic interactions through Phe276. In addition, even though a single amino acid change in EstE1_V274A _and EstE1_F276A _did not convert them to monomers, the combination of these two mutations in EstE1_V274A/F276A _abolished its ability to form dimers, suggesting the hydrophobic interactions involving both Val274 and Phe276 are important for EstE1 dimerization. Likewise, native PAGE analysis revealed a faster electrophoretic mobility of EstE1_F276E _and EstE1_V274A/F276A _compared to that of wild-type EstE1 (data not shown). In contrast, the EstE1 mutant proteins with Ala substitution of Arg270 or Asp295 (EstE1_R270A _and EstE1_E295A_), and the mutant with Ala substitution on both of these residues (EstE1_R270A/E295A_) were still able to dimerize, indicating salt bridges at the both ends of the hydrophobic interface are not critical for EstE1 dimerization.

### The relationship between the dimerization and thermostability of EstE1

**Figure 5:**
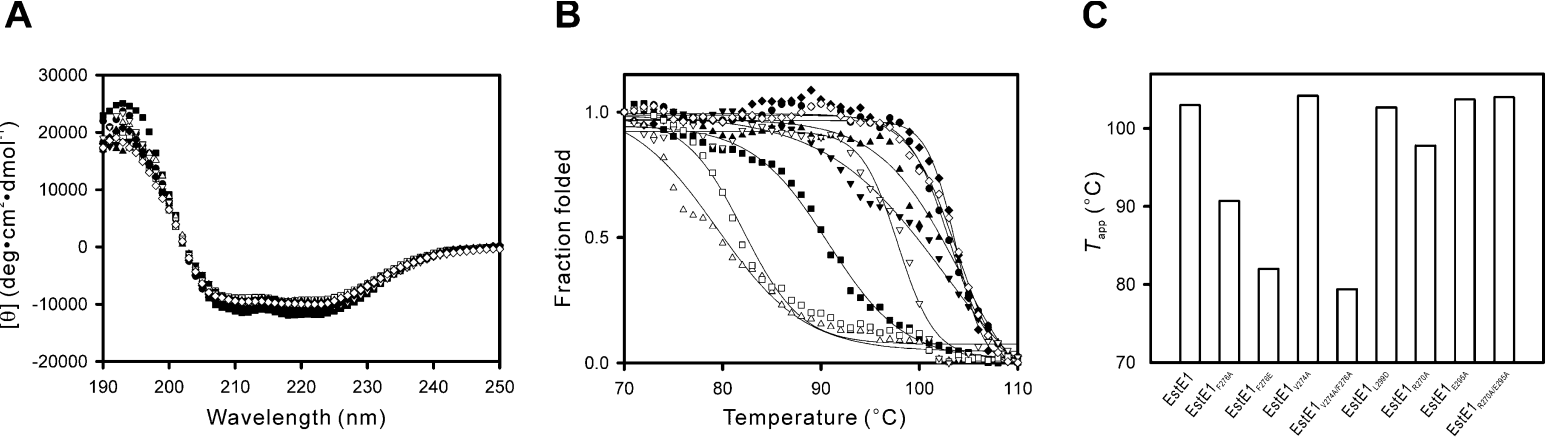
**Thermal denaturation profiles of wild-type and mutant EstE1**. (A) Far-UV CD spectra of wild-type EstE1 (●), EstE1_F276A _(■), EstE1_F276E _(□), EstE1_V274A _(▲), EstE1_V274A/F276A _(▯), EstE1_L299D _(▼), EstE1_R270A _(▯), EstE1_E295A _(▯), and EstE1_R270A/E295A _(▯) are plotted as molar ellipticity versus wavelength. (B) Thermal denaturation profiles of wild-type and mutant EstE1. Changes in molar ellipticity at 222 nm at a scan rate of 1°C/min in the temperature range of 70 to 110°C were measured, and the fractions folded are plotted. (C) Apparent transition temperatures of wild-type and mutant EstE1. *T*_app _values were estimated by fitting the data shown in (B), using the five-parameter sigmoid function from the curve-fitting program SIGMAPLOT. The inflection point was determined by numerical differentiation of the curves as described previously [8].

To investigate the relationship between the dimerization and thermostability of EstE1, we compared the thermostability of the EstE1 mutant proteins by thermal denaturation experiments using CD spectroscopy. CD spectra of all EstE1 mutants in the far-UV region at 20°C were very similar to that of wild-type EstE1 (Figure 5A), confirming that the integrity of secondary structure of the mutant proteins was not affected by the introduction of mutations. We then determined the apparent transition temperature (*T*_app_) [8,23] by monitoring changes in secondary structure content in the temperature range of 70 to 110°C (Figure 5B). As shown in Fig. 5C, the *T*_app _value for EstE1_F276E _and EstE1_V274A/F276A_, which converted to monomers (Figure 4), showed an approximate 20°C decrease in *T*_app_. This demonstrates that both Phe276 and Val274, which are important for intermolecular hydrophobic interactions, are critical for thermostability of EstE1. In contrast, mutations of the residues involved in interfacial salt bridges did not significantly decrease the *T*_app _value. The *T*_app _values for the EstE1_R270A_, EstE1_E295A_, EstE1_R270A/E295A_, and EstE1_Y250F _were estimated to be 97.8, 103.7°C, 104, and 98.2°C, respectively. None of these mutants lost their ability to form dimers (Figure 4), suggesting that hydrophobic interactions responsible for EstE1 dimerization are the main contributors to the thermostability of EstE1.

**Figure 6:**
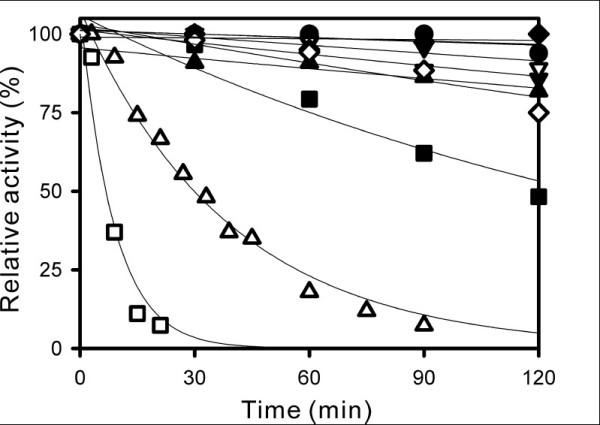
**Kinetic analysis of the thermostability of wild-type and mutant EstE1**. The enzymes (6.0 μM) of wild-type EstE1 (●), EstE1_F276A _(■), EstE1_F276E _(□), EstE1_V274A _(▲), EstE1_V274A/F276A _(▯), EstE1_L299D _(▼), EstE1_R270A _(▯), EstE1_E295A _(▯), and EstE1_R270A/E295A _(▯), in 20 mM potassium phosphate buffer (pH 7.0) were incubated at 80°C for the indicated times. Residual activities were then determined by measuring the amount of *p*-nitrophenol released by esterase-catalyzed hydrolysis. The activity of a non-incubated sample was defined as 100%

Kinetic analyses of the thermal stability of wild-type and mutant EstE1 proteins also support the notion that the thermostability of EstE1 correlates with its ability to form a dimer (Figure 6). By mutating the sites involved in the hydrophobic interaction between two molecules of EstE1, EstE1_F276E _and EstE1_V274A/F276A _dramatically lost their enzyme activities after incubation for 1 h at 80°C. The estimated half-life values of EstE1_F276E _and EstE1_V274A/F276A _were 6.5 and 29 min, respectively. In contrast, wild-type EstE1 showed no significant decrease in activity even after incubation for 2 h. EstE1_F276A_, which did not exist in a monomeric form, lost approximately 50% of its activity after incubation for 2 h at 80°C, suggesting that weaker hydrophobic interactions can be disrupted during heat denaturation. This result is consistent with the thermal denaturation pattern of EstE1_F276A_, displaying an earlier denaturation profile compared to wild-type EstE1 (Figure 5B). EstE1_L299D _appeared as a dimer (Figure 4) and retained a high thermostability, indicating that Leu299 is not detrimental for EstE1 dimerization and thermostability. Introduction of mutations to the residues involved in salt bridges at the EstE1 dimer interface did not induce conversion of EstE1 dimer to a monomer form (Figure 4). EstE1_R270A _and EstE1_R270A/E295A _displayed a slight decrease in their thermostability (over 75% activity after incubation for 2 h at 80°C), and EstE1_E295A _still maintained its activity (Figure 6). These results suggest that salt bridges are not the major contributor required for EstE1 dimerization and thermostability, whereas two hydrophobic interfacial spots, Val274 and Phe276 on the β8 strand of two molecules of EstE1, are critical for EstE1 dimerization and thermostability.

## Discussion

We determined the crystal structure of EstE1 at 2.1-Å resolution by the SAD method. EstE1 has a characteristic α/β hydrolase fold with nine alpha helices and an eight-stranded β-sheet. The residues Ser154, Asp251, and His281 of the catalytic triad are found in the canonical positions observed in a wide variety of α/β hydrolases [21]. Four members of the HSL family have been structurally characterized. They include an esterase from the mesophilic bacterium *Alcaligenes eutrophus *[24], the BFAE from the mesophilic bacterium *Bacillus subtilis *[20], and two thermophilic esterases, the EST2 from the thermophilic bacterium *Alicyclobacillus acidocaldarius *[18], and the AFEST from the hyperthermophilic archaeon *Archaeoglobus fulgidus *[19]. EstE1 is closer in structure to HSL-family thermostable esterases AFEST (Protein Data Bank (PDB) code 1JJI; 283 Cα atoms superimposed, root-mean-square deviation (rmsd) of 1.3 Å) and EST2 (PDB code 1EVQ; 280 Cα atoms superimposed, rmsd of 1.5 Å) than to mesophilic esterases, an esterase from *Alcaligenes eutrophus *(PDB code 1QLW; 196 Cα atoms superimposed, rmsd of 2.7 Å) and BFAE (PDB code 1JKM; 282 Cα atoms superimposed, rmsd of 2.0 Å).

EstE1 exists as a dimer through hydrophobic interactions and salt bridges (Figures 1 and 2). The thermostable esterase AFEST, which is most closely related with EstE1 both in structure and amino acid sequence, also was known to exist as dimers, and the intermolecular contacts of AFEST primarily consist of hydrogen bonds and salt bridges [19]. Our comparison of the structures of EstE1 and AFEST revealed that, although β-sheets of the core domain of these thermostable esterases are almost identically arranged, the overall content of secondary structure elements in EstE1 and AFEST show slightly different configurations. EstE1 consists of 39.5% helical and 16.7% β-strand conformations, while AFEST has 43.1% helical and 21.5% β-strand conformations [25]. This suggests that EstE1 has more turns and loops that might result in decreased thermostability of EstE1 because, in the order of mesophilic/thermophilic/hyperthermophilic proteins, gradually reduced dimensions in the loop regions are likely to presented [26]. Nevertheless, EstE1 displays a higher thermostability compared to AFEST. AFEST had approximately 50% residual activity after incubation for 30 min at 95°C with a half life of 26 min, whereas EstE1 still displayed > 95% residual activity under the same conditions [8,10]. This hyperthermostability of EstE1 is most likely due to strong hydrophobic interactions at the dimeric interface. Alignment of the amino acid sequences of EstE1 and AFEST revealed that the residues playing a key role in EstE1 dimerization differ from those of AFEST (Figures 2E and 3). The hydrophobic residues Val274, Phe276, and Leu299, which are involved in EstE1 dimerization, correspond to Val278, Tyr280, and Gln303 of AFEST, respectively. Unlike EstE1, AFEST cannot form strong hydrophobic interactions through these residues. AFEST dimers appear to be formed by hydrogen bonds (Tyr280_A_-Gln303_B _and Tyr280_B_-Gln303_A_) and weak hydrophobic interactions between Val278_A _and Val278_B_.

Because hydrophobic effect is considered to be a key feature that leads proteins to fold into its functional configuration [27,28], it also is assumed to be the major force involved in protein thermostabilization in thermal environments [29]. Kinetic analyses of the thermal stability of wild-type and mutant EstE1 proteins revealed that the elevated thermostability of EstE1 correlates with its ability to form dimers (Figures 4 and 6). The activity of EstE1_F276E _and EstE1_V274A/F276A_, which are present as a monomer, was dramatically diminished when they were incubated at 80°C. Although EstE1 retained its dimeric status upon mutation of either Val274 or Phe276 to Ala (Figure 4), mutation of both of these amino acids to Ala (EstE1_V274A/F276A_) destabilized the EstE1 and converted it to a monomer in solution. These results suggest that the intermolecular hydrophobic interactions through Val274 and Phe276 between two molecules of EstE1 largely contribute to the thermostability of EstE1. Mutation of either Val274 or Phe276 to Ala rendered the mutant proteins more sensitive to heat denaturation, with the latter mutation being more effective in destabilizing EstE1 dimers during the course of heat denaturation (Figure 5) and thermostability kinetic analysis (Figure 6). Our analysis of the β-sheet stability index (mean curvature value, which has a smaller value for a more stable residue in the β-sheet) [30] predicted the extent to which Val274 and Phe276 in β-strand 8 contributed to the stability of the intermolecular β-sheet bridges. The results showed that the mean curvature of Phe276 (0.059) was smaller than that of Val274 (0.119), suggesting that alteration of the more stable residue Phe276 on the β-strand could result in destabilization of EstE1 dimers by altering the β-strand conformation. Previously, mesophilic BFAE also was shown to form a dimer by a network of hydrogen bonds and ionic interactions [20], but detailed information was not provided. Our analysis of the dimeric interface of BFAE revealed that its dimerization is mediated mainly by the ionic interactions between side chains of each subunit (Glu306-Arg319, Glu353-Arg366, Arg357-Asp365 and Arg357-Asp358), and by the main chain-main chain hydrogen bonds at the dimeric interface (between the β8 strand of each subunit). Alignment of BAFE and EstE1 amino acid sequences shows that the hydrophobic residues Val274, Phe276, and Leu299 in EstE1 were substituted with the hydrophilic residues, Arg331, Asn333, and Arg357 in BFAE, respectively (Figure 3). BFAE exists as a dimeric form in solution [20], like EstE1, but the dimer interactions are quite distinct in that EstE1 dimer has tight hydrophobic interactions assisted by ionic interactions, while BFAE dimerization is mainly mediated by ionic interactions. Therefore, thermostability of EstE1 is probably explained by hydrophobic interaction-mediated dimerization not observed in one of its homologous mesophilic esterases, BFAE.

In our previous work, we compared the sequence and structure of esterases from thermophiles with that of their mesophilic counterparts to demonstrate that changes in the size or packing state of hydrophobic clusters in the cavity of EstE1 affect its thermostability. Likewise, we also identified ion-pair networks consisting of Arg61, Arg97, and Arg147, that contribute to the stabilization of EstE1 in thermal environments [8]. Mutation of Ala75, which is located in the hydrophobic cavity of EstE1, to Gly in EstE1_A75G _more effectively decreased the thermostability than other mutations of residues involved in intramolecular ion-pair network formation [8]. EstE1_A75G_, however, still exhibited ~75% residual activity after incubation for 30 min at 95°C [8], while mutation of Val274 and Phe276, which were identified in this work as the residues most critical for EstE1 thermostability, resulted in no detectable level of activity and ~25% residual activity, respectively, after incubation for 30 min at 80°C (Figure 6). Together, these results underscore the importance of hydrophobic interactions-mediated dimerization in the hyperthermostability of EstE1. A recent analysis of structures and sequences of several hyperthermostable proteins proposed two possible mechanisms for thermostabilization of proteins, "structure-based" and "sequence-based" mechanisms of thermostability, which possibly explain an evolutionary strategy of thermophilic adaptation of proteins [31]. The authors found that hyperthermophilic archaea possibly used structure-based mechanisms to increase the thermostability of their proteins, and that the proteins were "*de novo*"-designed to be more compact than their mesophilic counterparts. Indeed, EstE1 has a relatively higher number of van der Walls contacts per residue (126 and 125 for dimer and monomer, respectively, which is comparable number obtained with various hyperthermophilic proteins [31]) compared to the mesophilic BFAE (124 and 122 van der Walls interactions for dimer and monomer, respectively). Thus, compactness of EstE1 structure provides a plausible explanation for its hyperthermostability. Thermostability determinants of EstE1 identified in this study and in our previous work [5] altogether suggest that EstE1 might be originated from a hyperthermophilic archaeon because its thermostability mainly relies on the "structure-based' mechanism as described above. Our experiments-based prediction of the origin of EstE1 is consistent with the previous data showing that EstE1 is more closely related to hyperthermophilic archaea-originated HSL-family esterases/lipases, as demonstrated by phylogenetic tree analysis [5].

## Conclusion

Our structural analysis of the EstE1 dimer structure identified hydrophobic interactions and salt bridges that contributed to the formation of EstE1 dimers. Site-directed mutagenesis analysis revealed that hydrophobic interactions through Val274 and Phe276 on the β8 strand at dimer interface contribute to the formation of EstE1 dimers and their hyperthermostability. Our data along with the previous ones [8] suggest that the hyperthermostability of EstE1 is likely the result of a delicate balance between several factors including ion-pair networks and hydrophobic interactions. Nevertheless, the hyperthermostability of EstE1 seems to be achieved mainly by its dimerization through hydrophobic interactions, rather than intermolecular and intramolecular ion-pair networks even though they additively contribute to further stabilization of EstE1. EstE1 therefore might be evolutionally adapted to thermal environment by its intrinsic structural ability to form compact and stable dimers [31].

## Methods

### Plasmids

The EstE1 esterase expression vector pET-22b(+)-estE1 has been previously described [8]. EstE1 mutants were constructed by site-directed mutagenesis with two sequential rounds of PCR using oligonucleotides containing the target mutation as described previously [8]. Primer sequences are available upon request. The PCR products were digested with *Nde*I and *Not*I and ligated with pET-22b(+) (Novagen) to obtain the expression vectors for EstE1 mutant proteins. The presence of the correct mutation was verified by DNA sequencing.

### Expression and purification of EstE1 proteins

EstE1 was expressed in *E. coli *B834(DE3) cells (Novagen) transformed with pET-22b(+)-estE1 [8]. Cells were cultured at 37°C in liquid M9 medium containing 19 amino acids and selenomethionine (SeMet) to express SeMet-EstE1 protein with a C-terminal six-histidine tag. Protein expression was induced at 25°C for 12 h by addition of 1 mM isopropyl-β-D-thiogalactopyranoside. Cellular extracts were heat-treated at 80°C for 10 min, and SeMet-EstE1 was purified by affinity chromatography using Ni-charged Chelating Sepharose Fast Flow resin (Amersham Biosciences) as described previously [32]. Purified EstE1 was stored in 50 mM sodium phosphate pH 8.0, 300 mM NaCl, 10 mM β-mercaptoethanol, and 10% glycerol, and was further concentrated to approximately 10 mg/ml for crystallization. For biochemical studies of wild-type and mutant EstE1, *E. coli *BL21(DE3) cells (Novagen) were transformed with each plasmid construct and recombinant protein was purified from non-heat treated cellular extracts as described previously [8].

### Crystallization and data collection

Crystallization of EstE1 was performed by the hanging-drop vapor diffusion method as described previously [32]. Briefly, SeMet crystals suitable for data collection were obtained by mixing 1.5 μl of protein sample and 1.5 μl of reservoir solution (0.1 M Bis-Tris, 0.2 M ammonium sulfate, 1 M lithium sulfate, pH 6.5) and were equilibrated against 1 ml of reservoir solution. Before mounting, the crystals were transferred and soaked in cryo-protective solution containing 15% ethylene glycol and reservoir solution. Selenium X-ray single-wavelength anomalous dispersion (SAD) data of the crystals were collected on a Bruker Proteum 300 CCD detector at the 6B and 4 MX beamlines of Pohang Light Source in Korea. Data were processed with DENZO and SCALEPACK from the HKL package [33,34].

### Structure determination and refinement

The structure of EstE1 was solved by SAD method. Phasing and automatic tracing was performed using the SOLVE/RESOLVE package [35]. The expected 8 selenium sites were identified using SOLVE, and density modification and model building was performed using RESOLVE. A tracing of C_α _for the N-terminal residues (Leu17-Ala20) and the C-terminal residues (Glu295-Pro310) was conducted manually using the program O, and most residues fit into the electron density map [35]. Crystal structure was refined using the program CNS [16]. The quality of the model was assessed with the program PROCHECK [36]. van der Waals interactions were calculated using CCP4 program suite CONTACT [37]. Only the contact distances between 2.5–5.0 Å were considered. Figures 1 and 2 were prepared using the PyMOL 0.93 software. The atomic coordinates and structure factors have been deposited in the Protein Data Bank, Research Collaboratory for Structural Bioinformatics, Rutgers University, New Brunswick, NJ, with accession code 2C7B.

### Gel filtration chromatography

Esterase-containing fractions from the Ni-affinity column were combined and dialyzed against gel filtration column buffer (50 mM Tris-HCl, 150 mM NaCl, 1 mM DTT, 0.5 mM EDTA, pH 7.8), and then subjected to GFC using a Superdex 200 column (Amersharm Biosciences). Proteins were eluted from the column at a flow rate of 0.5 ml/min. Eluates were monitored with a UV detector at a wavelength of 280 nm. The column was calibrated using known molecular weight standards to construct a calibration graph from which the molecular weights of the eluted proteins were calculated.

### Circular dichroism

Circular dichroism (CD) spectra were collected on a J-810 spectropolarimeter (Jasco, Tokyo, Japan) equipped with a Peltier temperature-control system (Model PTC-423S) as described previously [8]. Thermal denaturation curves were obtained by monitoring the change in the molar ellipticity at 222 nm at a scan rate of 1°C/min in the temperature range of 70 to 110°C. Because of irreversible unfolding of EstE1 proteins under these CD spectroscopy conditions, apparent transition temperature (*T*_app_) values were estimated by fitting the data using the five-parameter sigmoid function as described previously [8,23].

### Heat inactivation and enzyme assays

Thermostability was analyzed by measuring the residual activities after incubating the enzymes (6.0 μM in 20 mM potassium phosphate buffer, pH 7.0) at 80°C for various times. Esterase activity was determined by measuring the amount of *p*-nitrophenol released by hydrolysis of *p*-nitrophenyl caproate as described previously [5].

## Abbreviations

CD, circular dichroism; GFC, gel filtration chromatography; HSL, hormone-sensitive esterase/lipase; 3D, three-dimensional

## Authors' contributions

JSB and JKR contributed equally to this work and should be considered as co-first authors. JKR made the original clone, designed the mutants, and performed biochemical experiments. JSB and DUK purified, crystallized the SeMet form of EstE1, collected and processed the diffraction data, refined, and analyzed the structure. JSB and JKR prepared the initial draft of the manuscript. NDK and JY provided significant bioinformatics input and participated in designing of EstE1 mutants. JWO and HSC conceived of the study, coordinated all the components of the project, and prepared final manuscript with additional input from EK. All authors have read and approved the final manuscript.

## References

[B1] Bornscheuer UT (2002). Methods to increase enantioselectivity of lipases and esterases. Curr Opin Biotechnol.

[B2] Moore JC, Arnold FH (1996). Directed evolution of a para-nitrobenzyl esterase for aqueous-organic solvents. Nat Biotechnol.

[B3] Altaner C, Saake B, Tenkanen M, Eyzaguirre J, Faulds CB, Biely P, Viikari L, Siika-aho M, Puls J (2003). Regioselective deacetylation of cellulose acetates by acetyl xylan esterases of different CE-families. J Biotechnol.

[B4] Jaeger KE, Dijkstra BW, Reetz MT (1999). Bacterial biocatalysts: molecular biology, three-dimensional structures, and biotechnological applications of lipases. Annu Rev Microbiol.

[B5] Rhee JK, Ahn DG, Kim YG, Oh JW (2005). New thermophilic and thermostable esterase with sequence similarity to the hormone-sensitive lipase family, cloned from a metagenomic library. Appl Environ Microbiol.

[B6] Haki GD, Rakshit SK (2003). Developments in industrially important thermostable enzymes: a review. Bioresour Technol.

[B7] Arpigny JL, Jaeger KE (1999). Bacterial lipolytic enzymes: classification and properties. Biochem J.

[B8] Rhee JK, Kim DY, Ahn DG, Yun JH, Jang SH, Shin HC, Cho HS, Pan JG, Oh JW (2006). Analysis of the thermostability determinants of hyperthermophilic esterase EstE1 based on its predicted three-dimensional structure. Appl Environ Microbiol.

[B9] Hotta Y, Ezaki S, Atomi H, Imanaka T (2002). Extremely stable and versatile carboxylesterase from a hyperthermophilic archaeon. Appl Environ Microbiol.

[B10] Manco G, Giosue E, D'Auria S, Herman P, Carrea G, Rossi M (2000). Cloning, overexpression, and properties of a new thermophilic and thermostable esterase with sequence similarity to hormone-sensitive lipase subfamily from the archaeon Archaeoglobus fulgidus. Arch Biochem Biophys.

[B11] Rhee JK, Kim DY, Ahn DG, Yun JH, Jang SH, Shin HC, Cho HS, Pan JG, Oh JW (2006). Analysis of the Thermostability Determinants of Hyperthermophilic Esterase EstE1 Based on Its Predicted Three-Dimensional Structure. Appl Environ Microbiol.

[B12] Vonrhein C, Bonisch H, Schafer G, Schulz GE (1998). The structure of a trimeric archaeal adenylate kinase. J Mol Biol.

[B13] Singleton M, Isupov M, Littlechild J (1999). X-ray structure of pyrrolidone carboxyl peptidase from the hyperthermophilic archaeon Thermococcus litoralis. Structure.

[B14] Kirino H, Aoki M, Aoshima M, Hayashi Y, Ohba M, Yamagishi A, Wakagi T, Oshima T (1994). Hydrophobic interaction at the subunit interface contributes to the thermostability of 3-isopropylmalate dehydrogenase from an extreme thermophile, Thermus thermophilus. Eur J Biochem.

[B15] Kelly CA, Nishiyama M, Ohnishi Y, Beppu T, Birktoft JJ (1993). Determinants of protein thermostability observed in the 1.9-A crystal structure of malate dehydrogenase from the thermophilic bacterium Thermus flavus. Biochemistry.

[B16] Tanaka Y, Tsumoto K, Yasutake Y, Umetsu M, Yao M, Fukada H, Tanaka I, Kumagai I (2004). How oligomerization contributes to the thermostability of an archaeon protein. Protein L-isoaspartyl-O-methyltransferase from Sulfolobus tokodaii. J Biol Chem.

[B17] Brunger AT, Adams PD, Clore GM, DeLano WL, Gros P, Grosse-Kunstleve RW, Jiang JS, Kuszewski J, Nilges M, Pannu NS, Read RJ, Rice LM, Simonson T, Warren GL (1998). Crystallography & NMR system: A new software suite for macromolecular structure determination. Acta Crystallogr D Biol Crystallogr.

[B18] Ollis DL, Cheah E, Cygler M, Dijkstra B, Frolow F, Franken SM, Harel M, Remington SJ, Silman I, Schrag J (1992). The alpha/beta hydrolase fold. Protein Eng.

[B19] De Simone G, Galdiero S, Manco G, Lang D, Rossi M, Pedone C (2000). A snapshot of a transition state analogue of a novel thermophilic esterase belonging to the subfamily of mammalian hormone-sensitive lipase. J Mol Biol.

[B20] De Simone G, Menchise V, Manco G, Mandrich L, Sorrentino N, Lang D, Rossi M, Pedone C (2001). The crystal structure of a hyper-thermophilic carboxylesterase from the archaeon Archaeoglobus fulgidus. J Mol Biol.

[B21] Wei Y, Contreras JA, Sheffield P, Osterlund T, Derewenda U, Kneusel RE, Matern U, Holm C, Derewenda ZS (1999). Crystal structure of brefeldin A esterase, a bacterial homolog of the mammalian hormone-sensitive lipase. Nature Structural Biology.

[B22] Heikinheimo P, Goldman A, Jeffries C, Ollis DL (1999). Of barn owls and bankers: a lush variety of [alpha]/[beta] hydrolases. Structure.

[B23] Nardini M, Dijkstra BW (1999). [alpha]/[beta] Hydrolase fold enzymes: the family keeps growing. Current Opinion in Structural Biology.

[B24] Duy C, Fitter J (2005). Thermostability of irreversible unfolding alpha-amylases analyzed by unfolding kinetics. J Biol Chem.

[B25] Bourne PC, Isupov MN, Littlechild JA (2000). The atomic-resolution structure of a novel bacterial esterase. Structure.

[B26] Thompson MJ, Eisenberg D (1999). Transproteomic evidence of a loop-deletion mechanism for enhancing protein thermostability. Journal of Molecular Biology.

[B27] Vieille C, Zeikus GJ (2001). Hyperthermophilic enzymes: sources, uses, and molecular mechanisms for thermostability. Microbiol Mol Biol Rev.

[B28] Dill KA (1990). Dominant forces in protein folding. Biochemistry.

[B29] Jaenicke R, Lilie H (2000). Folding and association of oligomeric and multimeric proteins. Adv Protein Chem.

[B30] Jaenicke R, Bohm G (1998). The stability of proteins in extreme environments. Curr Opin Struct Biol.

[B31] Koh E, Kim T, Cho HS (2006). Mean curvature as a major determinant of beta-sheet propensity. Bioinformatics.

[B32] Berezovsky IN, Shakhnovich EI (2005). Physics and evolution of thermophilic adaptation. Proc Natl Acad Sci U S A.

[B33] Byun JS, Rhee JK, Kim DU, Oh JW, Cho HS (2006). Crystallization and preliminary X-ray crystallographic analysis of EstE1, a new and thermostable esterase cloned from a metagenomic library. Acta Crystallograph Sect F Struct Biol Cryst Commun.

[B34] Terwilliger TC (2001). Maximum-likelihood density modification using pattern recognition of structural motifs. Acta Crystallogr D Biol Crystallogr.

[B35] Terwilliger TC, Berendzen J (1997). Bayesian correlated MAD phasing. Acta Crystallogr D Biol Crystallogr.

[B36] Jones TA, Zou JY, Cowan SW, Kjeldgaard (1991). Improved methods for building protein models in electron density maps and the location of errors in these models. Acta Crystallogr A.

[B37] Laskowski RA MAMW (1993). PROCHECK: a program to check the stereochemical quality of protein structures. J Appl Crystallogr.

[B38] (1994). The CCP4 suite: programs for protein crystallography. Acta Crystallogr D Biol Crystallogr.

